# DNAGear- a free software for *spa* type identification in *Staphylococcus aureus*

**DOI:** 10.1186/1756-0500-5-642

**Published:** 2012-11-19

**Authors:** Faroq AL-Tam, Anne-Sophie Brunel, Nicolas Bouzinbi, Philippe Corne, Anne-Laure Bañuls, Hamid Reza Shahbazkia

**Affiliations:** 1 Universidade do Algarve, DEEI-FCT, Faro, Portugal; 2 UMR MIVEGEC IRD 244-CNRS 5290-Universités Montpellier 1 et 2, Institut de Recherche pour le Développement, 34394 Montpellier, France; 3 Centre Hospitalier Régional Universitaire de Montpellier, Service de Réanimation Médicale, Hôpital Gui de Chauliac, Université Montpellier 1, 34295 Montpellier cedex 5, France

## Abstract

**Background:**

*Staphylococcus aureus* is both human commensal and an important human pathogen, responsible for community-acquired and nosocomial infections ranging from superficial wound infections to invasive infections, such as osteomyelitis, bacteremia and endocarditis, pneumonia or toxin shock syndrome with a mortality rate up to 40%. *S. aureus* reveals a high genetic polymorphism and detecting the genotypes is extremely useful to manage and prevent possible outbreaks and to understand the route of infection. One of current and expanded typing method is based on the X region of the *spa* gene composed of a succession of repeats of 21 to 27 bp. More than 10000 types are known. Extracting the repeats is impossible by hand and needs a dedicated software. Unfortunately the only software on the market is a commercial program from Ridom.

**Findings:**

This article presents DNAGear, a free and open source software with a user friendly interface written all in Java on top of NetBeans Platform to perform *spa* typing, detecting new repeats and new *spa* types and synchronizing automatically the files with the open access database. The installation is easy and the application is platform independent. In fact, the SPA identification is a formal regular expression matching problem and the results are 100% exact. As the program is using Java embedded modules written over string manipulation of well established algorithms, the exactitude of the solution is perfectly established.

**Conclusions:**

DNAGear is able to identify the types of the *S. aureus* sequences and detect both new types and repeats. Comparing to manual processing, which is time consuming and error prone, this application saves a lot of time and effort and gives very reliable results. Additionally, the users do not need to prepare the forward-reverse sequences manually, or even by using additional tools. They can simply create them in DNAGear and perform the typing task. In short, researchers who do not have commercial software will benefit a lot from this application.

## Findings

### Background

Since recent decades, the epidemiological situation has changed: high prevalence of hospital-acquired methicillin-resistant *Staphylococcus aureus* (MRSA) in some countries and hospital units, outbreaks of community-acquired MRSA unrelated to the hospital, emergence of *S. aureus* from pigs transmitted to humans with colonization and some cases of human infection, emergence of MRSA with intermediate susceptibility to glycopeptides (GISA and heteroVISA) and description of strains resistant to vancomycin (VRSA) or linezolid, and the emergence of virulent strains responsible for shock toxinic, necrotizing pneumonia with a high mortality. Molecular typing is essential to detect the genetic polymorphism at microscale for molecular epidemiology study, especially in outbreak investigations, but also at a macroscale for phylogenetic and population-based analyses. Numerous molecular typing methods have been developed for *S. aureus*, including Pulsed-Field Gel Electrophoresis (PFGE), MultiLocus Sequence Typing (MLST), but also typing based on sequence polymorphisms of the following loci: the Staphylococcal Cassette Chromosome *mecA* (SCC*mec*), the accessory gene regulator (*agr*) and the X region encoding protein A (*spa* gene). In the last ten years, the *spa*-typing based on the X region of the protein A gene has become a standard method for *S. aureus*, see examples in [1-4]. The X region of *spa* gene is composed of variable numbers of repeats of 21 to 27 base pairs (24-bp repeat being the most common one). The polymorphism of this region is based on the deletions or duplications of these repeats and punctual mutations. Each unique sequence of repeats is defined by a value, and a unique combination of values identifies a *spa*-type. Numerous papers showed the usefulness of this region to characterize both micro- and macro-variation in *S. aureus*[1-8]. Nowadays more than 500 repeats and more than 10000 *spa*-types were described according to the sequence and organization of repeats, considering repeat polymorphism, association of repeats and number of repeats. To permit a global approach and to define a uniform code terminology of *spa*-repeats and -types, [9] developed the software Ridom StaphType that synchronizes either directly via the http-protocol or file based (*e.g*. via e-mail) with an accompanying SpaServer that functions as operative source for all new *spa*-repeat and -types codes. In this context, our objective was to provide a free and open source software synchronized with Ridom StaphType public database to determine from the raw *spa* sequence the repeats and types according to those already described in SpaServer and to identify the new ones. The X region of each *S. aureus* isolate was amplified by PCR as described by [6].

### Implementation

*Spa* sequence typing involves three main operations: Identifying sequence type, detecting new types and discovering new repeats. DNAGear is an application that is written in Java and built on top of NetBeans Platform which performs all of these operations. It offers both, an easy environment for users to work, and a modular underlying for developers for further improvements. In addition to these operations, the sequence forward-reverse alignment task is supported by the application.

#### File Format

**Figure 1:**
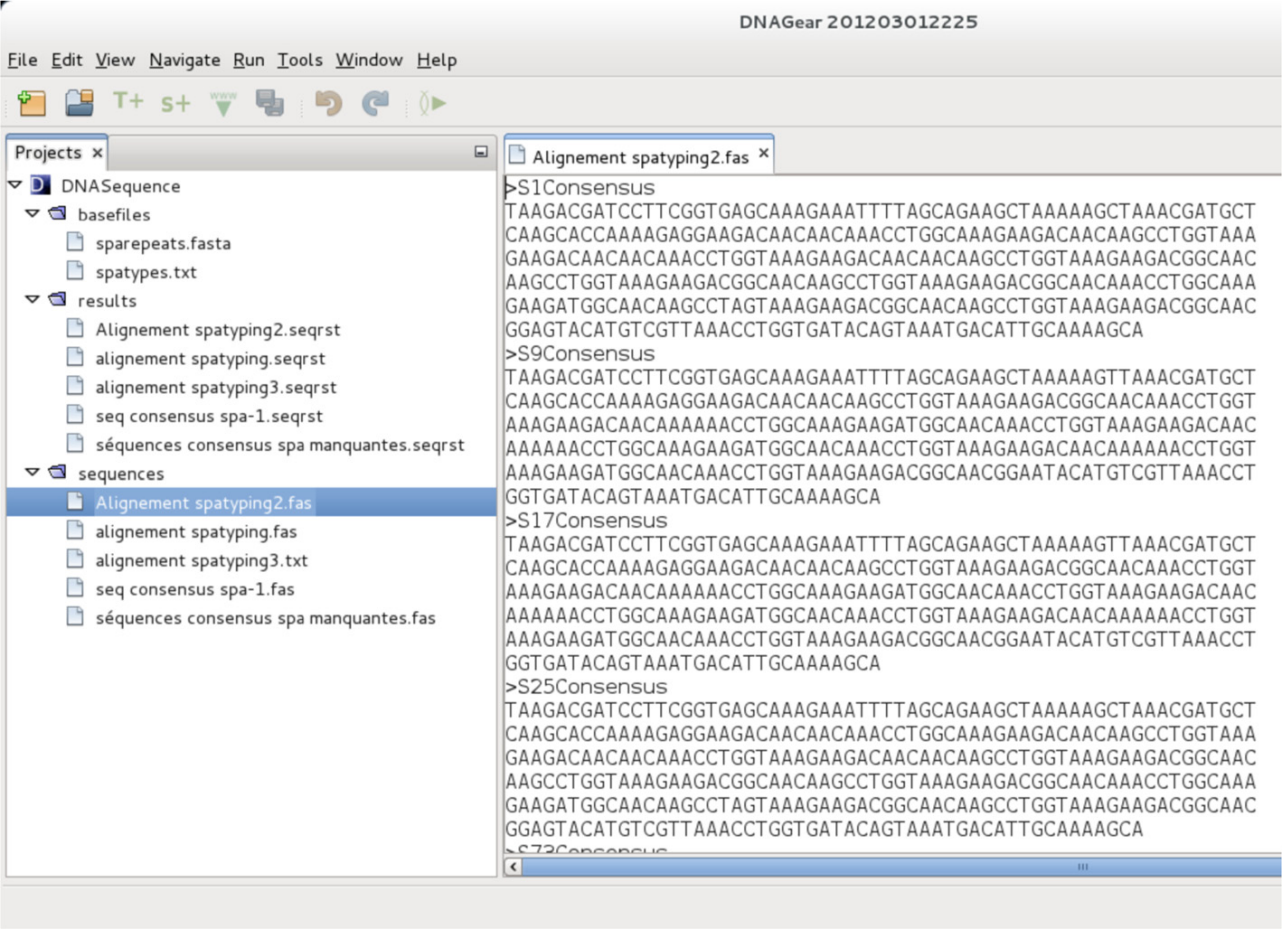
**DNAGear graphical user interface.** DNAGear main window. Project panel in the left, the menu and tool bar are at the top, and the working space (where the user can view and edit the files) is in the right.

**Figure 2:**
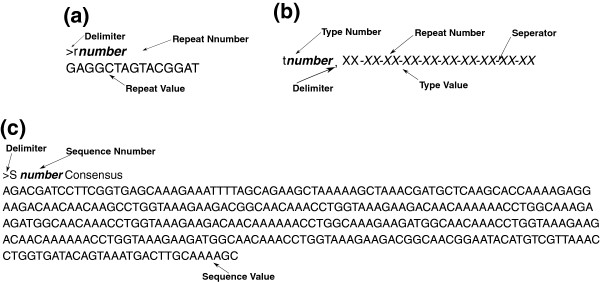
**Input file’s formats.a**- repeat entry format, **b**- type entry format and **c**- sequence entry format. The (XX) are two numerical decimal digits representing a repeat number.

DNAGear is a project-based application, Figure 1, in which the user can create multiple projects. Each project has input and output text files. Input files are: *repeats*, *types*, and *sequences* files. Each file has a set of entries. Each entry has name and value. The repeat’s value entry is a short DNA sequence, while the type’s one is a set of repeats’ numbers (part of the repeat name). Examples of these entries are shown in Figure 2, [see Additional file 1 also]. The output files are the processed sequences. Each entry in the output file has the original sequence entry followed by its type and the new repeats if exist in the sequence, [see Additional file 2].

#### Files Processing

*Sequence Typing and New Types and Repeats Detection:* To find a sequence type, for each sequence in the sequences’ file, after sorting the repeats according to their values’ lengths, do the following: 

1. For each repeat in the repeats’ file, find and replace each repeat’s value by the repeat’s number.

2. Use the regular expression “[-∖d∖d]+” to collect these numbers from the string resulted from step 1. The result is a hexadecimal-like string of numbers, see type value in Figure 2.

3. Look up the found result from step 2 in the types’ file to find the sequence type. If no type is found, then this type is marked as *new type* in the result file.

Please note that, the “∖d” in the regular expression “[-∖d∖d]+” can be rewritten as “[0-9]”. The “+” sign at the end of this expression means that we are looking for 1 or more repetition of two digits prefixed by a dash (“[-∖d∖d]”).

To detect new repeats, the following regular expressions are used [6]: 

• “AAAGAAGA[ACGT-]{4}AA[ACGT-]{1}AA[ACGT-]{1}[ACGT-]{1,4}CC[ACGT-]{4}”

• “GAGGAAGA[ACGT-]{4}AA[ACGT-]{1}AA[ACGT-]{1}[ACGT-]{1,4}CC[ACGT-]{4}”

Any sub-sequence in the resulted sequence, from step 1 above, that complies with these regular expressions is marked as *new repeat* in the result file.

For more algorithmic convenience and better understanding, let us use lists instead of files. In this context, let $S={\left\{s_{i}\right\}}_{i=1}^{i=N}$, $R={\left\{r_{j}\right\}}_{j=1}^{j=M}$, and $T={\left\{t_{k}\right\}}_{k=1}^{k=L}$ be the sequences, repeats, and types lists, respectively. In addition, let us define *η*(*x*) and *ν*(*x*) as the functions of the name and value of *x*, respectively, where *x*∈{*s*,*r*,*t*}. Our objective is to assign a type for each *s* and to detect the new repeats *R*^∗^ and types *T*^∗^ in *S*.

Before explaining the proposed method, let us denote by *τ*(*s*) to the type of a given sequence *s*. The algorithm of performing the *spa* typing tasks is shown in Algorithm 1. In this algorithm, the function sort(*R*)sorts the repeats’ list *R* according to their values’ lengths, so the repeat of the longest value is tested in the beginning to assure that a complete repeat value is replaced by the *Replace* function. The Replace(*ν*(*r*),*ν*(*s*),*η*(*r*)) function searches and replaces the repeat’s value *ν*(*r*)in the sequence’s value *ν*(*s*)by the repeat’s name (only the number part of the name is used, see Figure 2) *η*(*r*). The backbone of the algorithm is the regular expression compiler (performed by Java built-in well-established classes), it is denoted here by the function MatchRegEx(*regexp*,*str*). This function finds any substring in the string *str* that fits the regular expression *regexp*. The function add(*element*,*list*) adds *element* (if is not existing) to *list*.

Algorithm 1. SEQUENCEPROCESSING(S, R, T)

*Forward-reverse Sequence Alignment:* This is an additional feature (see [10] for more details), which helps the user to align the sequences automatically. The input of this task composes two sequence files. The first one is called forward and the other is called reverse. Initially, the reverse sequences is complemented. To find the complementary of a sequence, flip the letters of the input sequences as the following: then reverse it so that, the left most letter becomes the right most one and vise-versa. Finally, a maximum match between the forward sequence and the reversed complementary sequence is searched to find the sequences’ junction region (middle overlap) and produce one bigger sequence composed of the union of the complementary and forward sequences. The overlap region is found, by repeatedly, removing one letter from the forward sequence and check if the reverse complementary sequence starts with the remaining part of the forward sequence, until a match is found or the whole forward sequence has no more letters.

• A↔T

• C↔G

#### DNAGear Architecture

DNAGear is composed of three main modules, each of them contains a set of Java classes. These modules are: 

• DNAProject: This module manages the projects. Multiple projects can be created and processed by the same application instance, each project contains three main folders: *sequences*, *basefiles*, and *results*. The *sequences* folder contains the sequence files to be processed. The *basefiles* folder contains the repeats and types files. Once the sequences are processed, each sequence will have a result file stored in the *results* folder.

• DNAWorkSpace: This module is responsible for managing the Graphical User Interface (GUI) components. These components include, the main toolbar, the menus and actions.

• SequenceProcessing: This is the core module, it implements the typing of the sequences and the detection of new types and repeats tasks. It is composed of different classes, each one handles a certain entity. For example, repeat class performs repeats loading operation from the repeats file and manages the creation of repeat entries’ list, to be then used during the typing process.

### Results and Discussion

DNAGear regular expression and string manipulation is based on well-developed and optimized classes offered by Java. Nevertheless, it was tested on more than 50 *S. aureus* sequences for sequence typing and on more than 300 sequences for forward-reverse alignment task. The results were verified manually. In terms of accuracy, they showed a definite accuracy of finding sequences types, and detecting new types and repeats. Additionally, The biologists who tested it, reported no problem in the software so far. Although, the only existing and commercial software (Ridom StaphType) has more graphical features, support database management and offers some other meta-statistical reports, it is closed source and platform dependent. Furthermore, its core sequence typing tasks are similar to DNAGear. On the other hand, DNAGear has the following positive points: 

• it is free and open source

• supports automatic alignment of the forward-reverse sequences.

• it is platform independent and is shaped with different installers suitable for different target platforms (Linux, Windows, and MacOS).

• it performs the three major aspects of typing process.

• files are managed in project folders, which can be added, edited, moved, renamed or deleted easily. For example, in addition to adding a sequence file by using the designed tool bar button, the user can add it by dragging and dropping the file from its location on the disk to the *sequences* folder in the project tree.

It can also be extended very easily because it is built on a set of modules, that are managed by a very good Java modules development environment (NetBeans Platform 7.1), which allows developers to add new features as needed without deep understanding of the code already designed.

### Conclusions

A *spa* typing application has been developed to be freely available to the academic purposes. It finds sequence types, new types, and new repeats in the *Staphylococcus aureus* sequences. Furthermore, it has a rich and easy-to-use GUI environment which allows users to deal with as many as they want of files managed in projects. It also supports the forward-reverse sequence alignment for sequence assembling task. For portability and installation easiness, the system is built in Java and on top of the NetBeans Platform; therefore, it can run on different machines with different platforms.

## Availability and requirements

• Project name: DNAGear

• Project home page:http://w3.ualg.pt/hshah/DNAGear/

• Operating system(s): Platform independent

• Programming language: Java and the GUI is designed on top of NetBeans Platform 7.1

• Other requirements: JRE 1.6 or higher

• License: None

• Any restrictions to use by non-academics: None

Additionally, DNAGear has different installers that support the target platform.

## Competing interests

The authors declare that, they have no competing interests.

## Authors contributions

All authors contributed to the development of DNAGear. AS. Brunel, N. Bouzinbi, P. Corne and AL Bañuls provided the biological background and tested the results during development. H.R Shahbazkia and F. AL-Tam designed and implemented the algorithm and tested the software. All authors read and approved the final manuscript.

## Supplementary Material

Additional file 1 **A sample sequence file.** A sample sequence file in plain text (.txt) for testing the application. The entries in the file have the same sequence formating described earlier. Click here for file

Additional file 2 **A sample result file.** A sample result file in plain text (.txt) obtained from processing the sample sequence file (Additional file 1). Click here for file
